# ELMERF: A deep-learning-assisted hydroponic RGB phenotyping framework for rice seedling salt-stress evaluation and genetic mapping

**DOI:** 10.1016/j.plaphe.2026.100258

**Published:** 2026-07-03

**Authors:** Yunluo Li, Xianghui Meng, Qiyun Xu, Chen Xiong, Ran Xu, Hao Lu, Xiaodong Bai, Zhuang Yang, Hongyan Liu

**Affiliations:** a Sanya National Center of Technology Innovation for Saline-Alkali Tolerant Rice, School of Tropical Agriculture and Forestry (Sanya Institute of Breeding and Multiplication), Hainan University, Sanya, 572025, China; b State Key Laboratory of Digital Medical Engineering, Key Laboratory of Biomedical Engineering of Hainan Province, School of Biomedical Engineering, Hainan University, Sanya, 572025, China; c Hainan State Farms Academy of Sciences Group Co., Ltd., Sanya, 572024, China; d School of Computer Science and Technology, Hainan University, Haikou, 570228, China; e Sanya National Center of Technology Innovation for Saline-Alkali Tolerant Rice, School of Breeding and Multiplication (Sanya Institute of Breeding and Multiplication), Hainan University, Sanya, 572025, China; f School of Artificial Intelligence and Automation, Huazhong University of Science and Technology, Wuhan, 430074, China

**Keywords:** Rice, Salt stress, Semantic segmentation, Deep learning, Genome-wide association analysis

## Abstract

Rice seedling salt-tolerance evaluation commonly relies on visual scoring or destructive assays, which are subjective, labor-intensive, and difficult to standardize for population-level analysis. This study developed a new deep-learning-assisted hydroponic RGB phenotyping framework for standardized salt-stress evaluation and genetic mapping in rice seedlings. The framework integrates controlled hydroponic cultivation, RGB imaging, RicePhenoSeg-assisted annotation and trait extraction, ELMERF-based semantic segmentation, and image-derived quantification of salt-induced shoot injury. Using this framework, we constructed the Rice Seedling-Salt RGB Dataset (RSSD), which contains green shoot tissues, yellow shoot tissues, roots, and background from hydroponically grown rice seedlings. Based on RSSD, ELMERF achieved a mean Intersection over Union of 51.4% and a mean Accuracy of 89.5%, outperforming nine representative segmentation models. We further defined shoot yellowing rate (SYR) as an image-derived quantitative trait describing visible salt-induced shoot injury. The framework was applied to 261 re-sequenced rice accessions for population-level phenotyping and genome-wide association analysis. Compared with standard evaluation score and seedling death rate, SYR showed a more continuous phenotypic distribution and detected 36 significant SNPs, including a major signal near the Saltol/OsHKT1; 5 region. Notably, 34 SYR-associated SNPs were not detected by conventional visual scores. Overall, this study provides a targeted hydroponic RGB phenotyping framework for standardized rice seedling salt-stress evaluation and genetic analysis.

## 1 Introduction

Rice (*Oryza sativa* L.) is one of the world's most important food crops [1]. In recent years, approximately one-third of global arable land has been affected by varying degrees of salinization, which has become a major constraint on agricultural productivity [2]. According to the FAO, nearly 1.4 billion hectares of land worldwide are already affected by salinity, and an additional 1.0 billion hectares are at risk of salinization [3]. The seedling stage is one of the most sensitive periods during the rice life cycle, and salt stress during this period can strongly inhibit early growth and subsequently affect yield formation [4]. Physiologically, salt stress disrupts osmotic balance and induces ionic toxicity, leading to reactive oxygen species accumulation and reduced photosynthetic efficiency [5]. These physiological disturbances also accelerate chlorophyll degradation, which is often visually manifested as the transition of shoot tissues from green to yellow [6]. Therefore, efficient and reliable phenotyping methods for assessing seedling-stage responses to salt stress are urgently needed to identify salt-tolerant genotypes and support the breeding of resilient rice varieties.

Traditional methods for assessing salt tolerance in rice have been based primarily on manual visual scoring [7]. Although these methods remain widely used, they are subjective, low-throughput, and difficult to standardize across large populations. In addition, seedling-stage responses to salt stress often form continuous gradients rather than discrete categories, so visual grades may mask subtle but meaningful differences among genotypes. This limits their suitability for large-scale, objective, and reproducible salt-tolerance evaluation.

With the development of image-based phenotyping technologies, researchers can now non-destructively quantify plant morphological and physiological responses using RGB, fluorescence, and other imaging modalities [8,9]. These approaches have enabled high-throughput screening of salt- and stress-tolerant genotypes at the rice seedling stage. At the algorithmic level, traditional image analysis methods mainly rely on color-space thresholds such as HSV or Lab and machine-learning methods such as support vector machines (SVMs) [10,11]. However, most of these approaches still focus on whole-plant traits or field vegetation segmentation. As a result, they are not well suited for extracting fine-grained quantitative phenotypes of visible salt injury.

This limitation becomes more pronounced in seedling salt-stress phenotyping, where visible injury is often expressed as a progressive loss of greenness in shoot tissues. Under salt stress, shoot yellowing usually develops as a continuous transition from green to yellow rather than as sharply separated classes. In hydroponically grown seedlings, image interpretation is further complicated by irregular leaf margins, mottled yellowing patterns, slender tissue structures, and the coexistence of different plant organs within the same whole-plant image. Therefore, high-resolution organ-level segmentation is needed to improve the quantification of visible shoot injury and to convert it into robust quantitative phenotypes for salt-tolerance evaluation.

More recently, deep learning-based methods, including CNN- and YOLO-based frameworks, have extended image-driven salt-stress analysis [12,13]. In crop phenotyping, semantic segmentation has been widely applied to plant organ extraction, canopy structure analysis, senescence monitoring, disease assessment, weed detection, and seedling trait measurement [[14], [15], [16], [17], [18], [19]]. These studies demonstrate the value of deep learning for extracting quantitative traits from complex plant images. Nevertheless, most existing applications focus on canopy-scale images, disease or weed scenarios, general vegetation segmentation, or conventional seedling structural traits such as plant height and root length. They have rarely addressed the fine-grained separation of green shoot tissues, yellowing shoot tissues, and roots in hydroponically grown rice seedlings under controlled salt stress. More importantly, accurate segmentation alone is insufficient for salt-tolerance studies. It must be further translated into biologically meaningful quantitative traits that can support phenotypic comparison and genetic analysis.

Because salt tolerance is a complex quantitative trait, its genetic dissection also depends strongly on phenotype quality. Genome-wide association analysis (GWAS) has been widely used to identify loci underlying agronomic and stress-response traits in rice [20]. However, their effectiveness is strongly influenced by the precision, continuity, and reproducibility of phenotypic measurements [21,22]. Compared with conventional visual scores or endpoint survival-related traits, image-derived shoot-level phenotypes may provide a higher-resolution and more objective description of seedling salt injury. Such phenotypes may therefore improve the detection of biologically meaningful genetic signals [23]. However, image-based studies of rice salt stress have mostly relied on whole-image classification or coarse phenotypic indicators. A systematic framework linking high-resolution shoot phenotyping, semantic segmentation, and GWAS in rice seedlings is still lacking.

To address these challenges, we established an integrated framework that combines standardized hydroponic cultivation, image acquisition, semantic segmentation, image-derived quantification of salt injury and GWAS. The main contributions of this study are as follows.

1) We developed a standardized indoor hydroponic RGB imaging system for rice seedlings under controlled salt stress. Building on the widely used seedling-stage salinity screening framework [24], this system was designed to improve the repeatability and stability of salt-stress evaluation by reducing environmental heterogeneity and subjective visual scoring.
2) We constructed a dedicated rice seedling salt-stress RGB dataset (RSSD) under standardized hydroponic and imaging conditions. This dataset provides whole-seedling RGB images with pixel-level annotations of green shoot tissue, yellow shoot tissue, roots, and background, supporting fine-grained segmentation and quantitative extraction of visible salt-injury traits.
3) The enhanced multiscale relation fusion (ELMERF) semantic segmentation model and the RicePhenoSeg software were developed for multi-organ segmentation and phenotypic extraction from whole-seedling images. Based on this framework, we defined shoot yellowing rate (SYR) as an image-derived quantitative trait for evaluating visible shoot injury under salt stress. We then applied SYR to a GWAS using 261 resequenced rice accessions to assess the potential of image-derived phenotypes for seedling-stage salt-tolerance evaluation and locus discovery in rice.

## 2 Materials and methods

### 2.1 Plant materials and hydroponic culture system

#### 2.1.1 Hydroponic system construction and optimization (Phase 1: stability assessment)

**Fig. 1:**
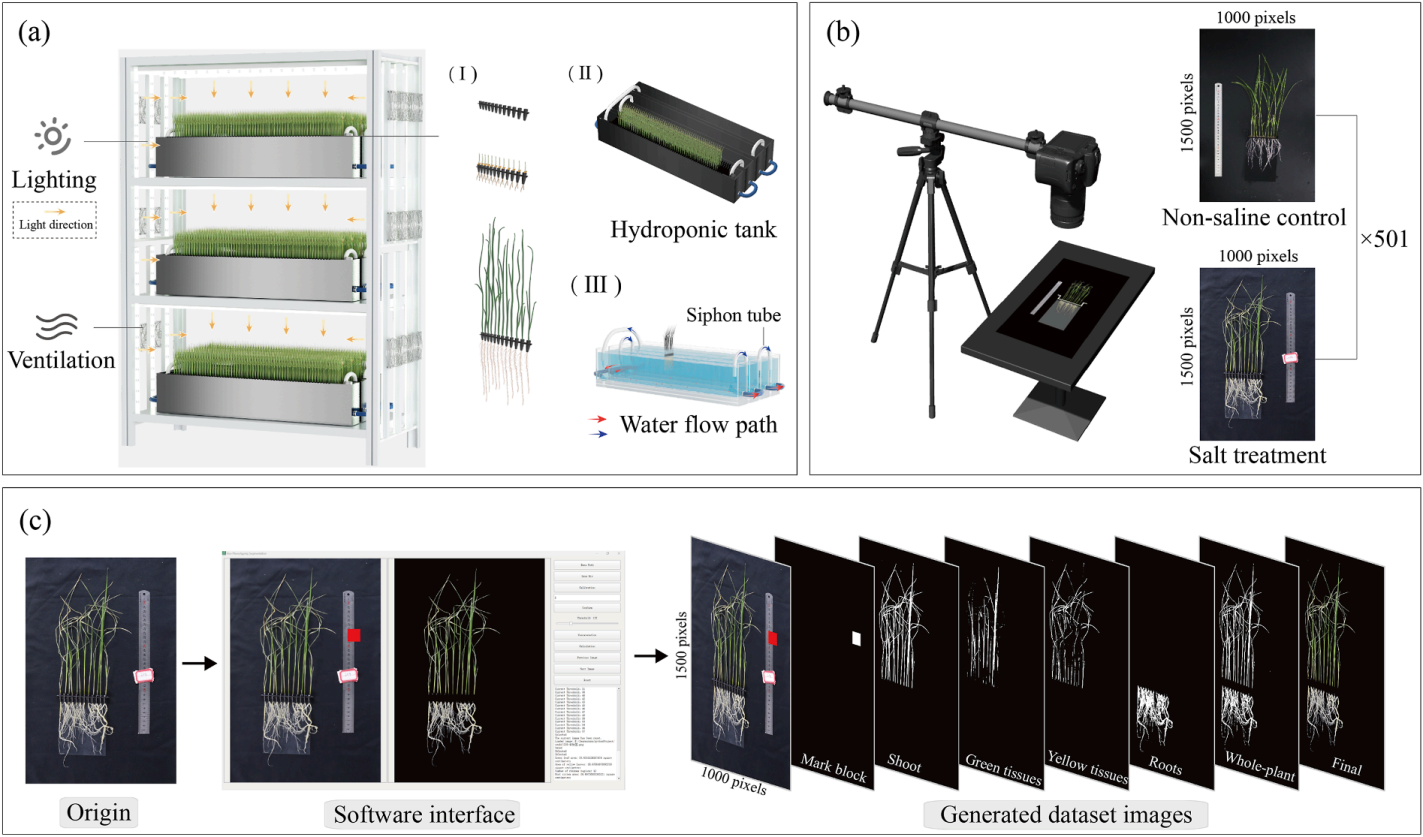
Workflow of dataset construction. (a) Hydroponic system for rice seedlings. (Ⅰ) Strip-type hydroponic container with seeds sown vertically in individual tubes. (Ⅱ) Internal structure of the hydroponic tank. (Ⅲ) Nutrient-solution circulation path driven by a water pump. (b) Rice seedling image acquisition. (c) RicePhenoSeg software for image segmentation and mask generation. The interface displays the raw whole-plant image and the corresponding segmentation result. It supports image loading, threshold adjustment, region correction, feature quantification, and mask export. Green shoot tissues, yellow shoot tissues, and roots are extracted for subsequent phenotypic analysis.

From 2023 to 2024, we established an indoor hydroponic cultivation system at the School of Breeding and Multiplication (Sanya), Hainan University, for stable and reproducible large-scale salt-stress evaluation. A total of 84 rice varieties, each with two replicates, were included in three independent optimization batches. The optimization focused on root-zone isolation, nutrient-solution circulation, light uniformity, and ventilation (Fig. 1a). Transparent partitions were installed beneath the strip-type hydroponic trays to prevent root entanglement among varieties (Fig. 1a.Ⅰ). Four hydroponic tanks were connected in series and operated with a circulation pump at a flow rate of 600 L h^−1^ to provide continuous nutrient flow, with nutrient solution pH = 6.0 (Fig. 1a.Ⅱ.Ⅲ). The LED lamp layout was adjusted to keep light intensity variation within the hydroponic area below 5%, with a maximum intensity of 30,800 lux (Fig. S1). The cultivation environment was maintained at 30°C with 70%-80% relative humidity. Fans installed on both sides of the system generated a horizontal airflow of approximately 1 m s^−1^, causing slight leaf movement. The final stability of the hydroponic system is shown in Fig. S2.

#### 2.1.2 Preparation of rice materials and salt-stress treatment (Phase 2: large-scale salt-tolerance evaluation)

After stabilization of the cultivation system, a total of 261 rice accessions were subjected to systematic salt-tolerance evaluation. All seeds underwent a standardized pretreatment procedure, including dormancy breaking at 50°C for 48 h, surface sterilization in 5% NaClO for 10 min, followed by rinsing with distilled water, soaking for 48 h, and germination in the dark at 30°C for 12 h. Germinated seeds with uniform radicle emergence were sown into 12-well strip-type hydroponic trays (one seed per well; one strip per accession) (Fig. 1a.Ⅰ). The water level was adjusted to just cover the seeds, and seedlings were grown in deionized water for 3 days to ensure uniform early seedling establishment after sowing. After this period, seedlings were transferred to Yoshida nutrient solution [7] and grown until the two-leaf stage. Salt treatment was then initiated by replacing the medium with Yoshida solution containing 0.8% NaCl and maintained for 7 days, with the solution refreshed every 3 days.

Immediately after salt treatment, seedling images were collected, and standard evaluation scores (SES) were visually recorded using the International Rice Research Institute standard evaluation system [7] for seedling-stage salt injury (Table S1). Seedlings were then transferred back to salt-free Yoshida solution for a 7-day recovery period. After recovery, seedlings with complete loss of greenness in the central leaf were recorded as dead. The seedling death rate (SDR) was calculated according to Eq. (1), where $N_{dead}$ and $N_{total}$ represent the number of dead seedlings and the total number of seedlings for each accession.

(1) $$SDR=\frac{N_{dead}}{N_{total}}\times 100\%$$

The resulting image data, SES, and SDR values jointly formed the dataset used for subsequent deep-learning-based phenotypic analysis and salt-tolerance evaluation.

#### 2.1.3 Image acquisition and dataset construction

A standardized imaging system was used to acquire rice seedling images (Fig. 1b). Images were captured with a Canon EOS 800D camera at a fixed distance of 60 cm. The camera settings were fixed at f/20, 1/15 s, and ISO 1600. LED lamps with a color temperature of 5500 K were used. A matte black plate was used as the background to reduce reflections. The lighting uniformity of the imaging area was maintained above 95%. After salt treatment, hydroponic trays containing seedlings were removed individually, and surface moisture was blotted off with absorbent paper. Each sample was photographed on the standardized background according to accession ID. One overhead RGB image was taken for each culture unit and used for shoot-level salt tolerance phenotyping. All images were linked to the corresponding plant number, standard evaluation score, and seedling death rate. The image dataset contained two subsets (Table 1). RSSD-A contained 501 images (excluding three cultivation units with no emergence) collected in January 2024. These images were used exclusively for segmentation model development. RSSD-B contained 261 images collected in September 2024. These images were used for image-derived phenotyping and GWAS validation. All images had a resolution of 1000 × 1500 pixels. After image acquisition, the self-developed segmentation software was used for organ-level segmentation and mask generation (Fig. 1c). The generated masks were used for model training, phenotypic extraction, and subsequent genetic analysis.

**Table 1:** The dataset was collected in two batches, which were used separately for model development and GWAS analysis.

Dataset	Collection Time	Image Size	Total	Train	Validation	Test	Category Proportions (%)				Usage
							Green shoot tissues	Yellow shoot tissues	Roots	Background	
RSSD-A	2024.01	1000 x 1500	501	400	50	51	2.19	0.42	0.89	96.5	Segmentation Model Development
RSSD-B	2024.09	1000 x 1500	261	∖	∖	∖	∖	∖	∖	∖	Phenotyping and GWAS Validation

During dataset construction, all 501 images were processed using the RPS software developed in this study, following unified category definitions and annotation rules. The annotated categories included green shoot tissues, yellow shoot tissues, roots, and background. All annotators received standardized training, and the annotation results were reviewed by professionals. The use of RPS helped reduce inconsistencies caused by manual operations during large-scale annotation.

ELMERF generated pixel-wise masks for three tissue classes: green shoot tissues, yellow shoot tissues, and roots. Within the segmented shoot region, the numbers of pixels assigned to green and yellow tissues were defined as green shoot pixels (GSP) and yellow shoot pixels (YSP), respectively. Based on these quantities, shoot yellowing rate (SYR) was calculated according to Eq. (2), where GSP + YSP represents the total number of segmented shoot pixels. SYR quantifies the proportion of visibly yellow tissues within the segmented shoot region.

(2) $$\text{SYR}=\frac{\text{YSP}}{\left(\text{GSP}+\text{YSP}\right)}\times 100\%$$

### 2.2 Framework of ELMERF

**Fig. 2:**
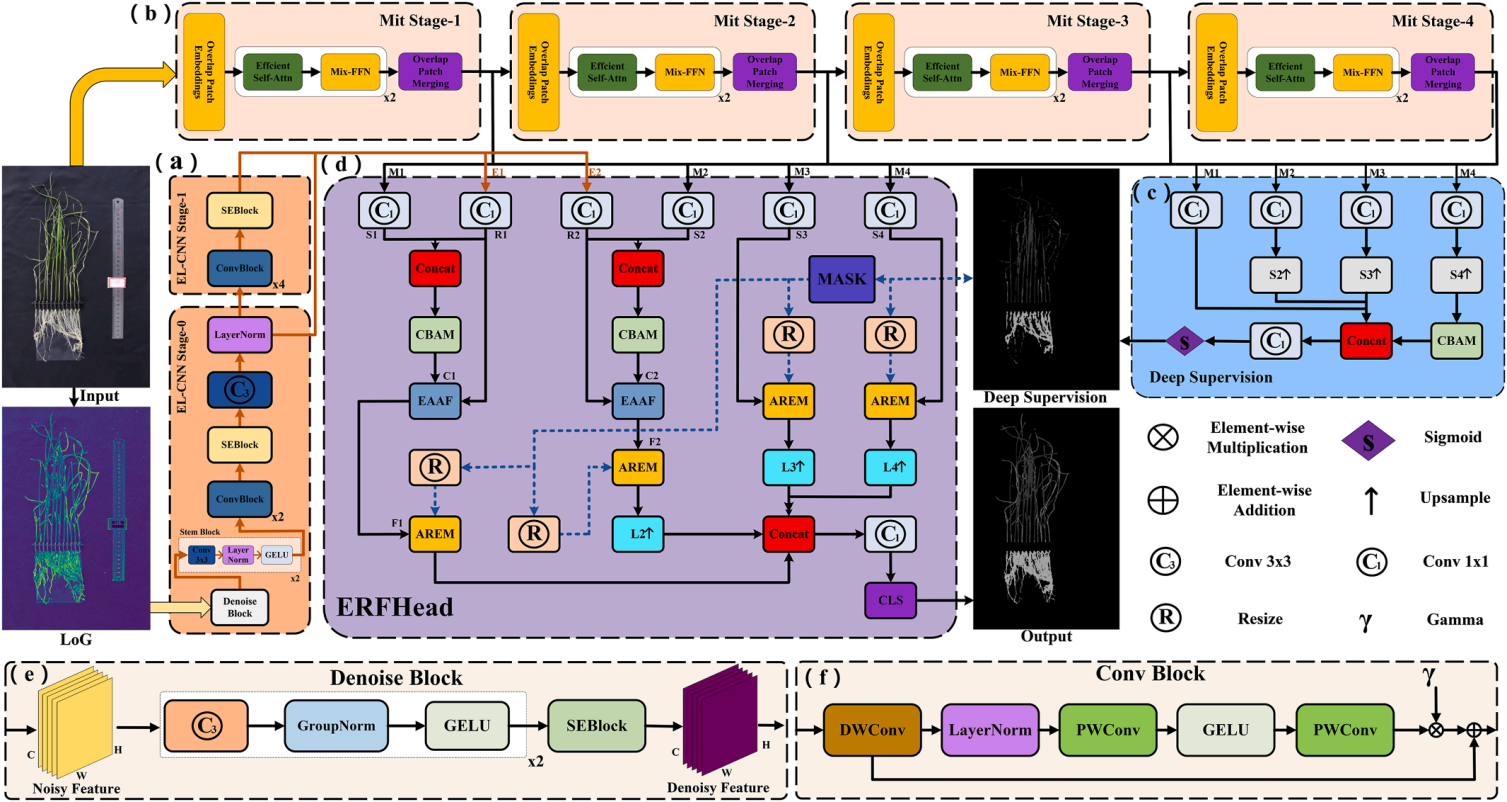
Overview of the ELMERF network architecture. The encoder contains a semantic feature extraction branch and a boundary feature extraction branch. The semantic branch uses MiT-B1 to generate multi-scale semantic features. The boundary branch uses a differentiable LoG filter, followed by denoising, SE, and convolution blocks, to extract edge features. The decoder adopts ERFHead to fuse semantic and boundary features. An intermediate supervision mask is generated from the semantic features and used to guide feature fusion. (a) Boundary feature extraction branch. (b) Semantic feature extraction branch. (c) Deep supervision module. (d) ERFHead decoder. (e) Denoising block. (f) Convolution block.

The Multi-scale Enhanced Relation Fusion (ELMERF) model proposed in this study follows an encoder-decoder architecture (Fig. 2). The encoder consists of a dual-branch backbone named Edge-LoG MixVision Transformer (EL-MViT), which is designed to extract semantic and boundary features. The Transformer branch adopts the MixTransformer (MiT) backbone from SegFormer [25] (Fig. 2b). It captures global contextual information and generates multi-scale semantic features. The Edge-guided LoG-CNN (EL-CNN) branch extracts fine-grained boundary details. The encoder outputs multi-scale feature maps that provide the decoder with complementary semantic and boundary information.

**Fig. 3:**
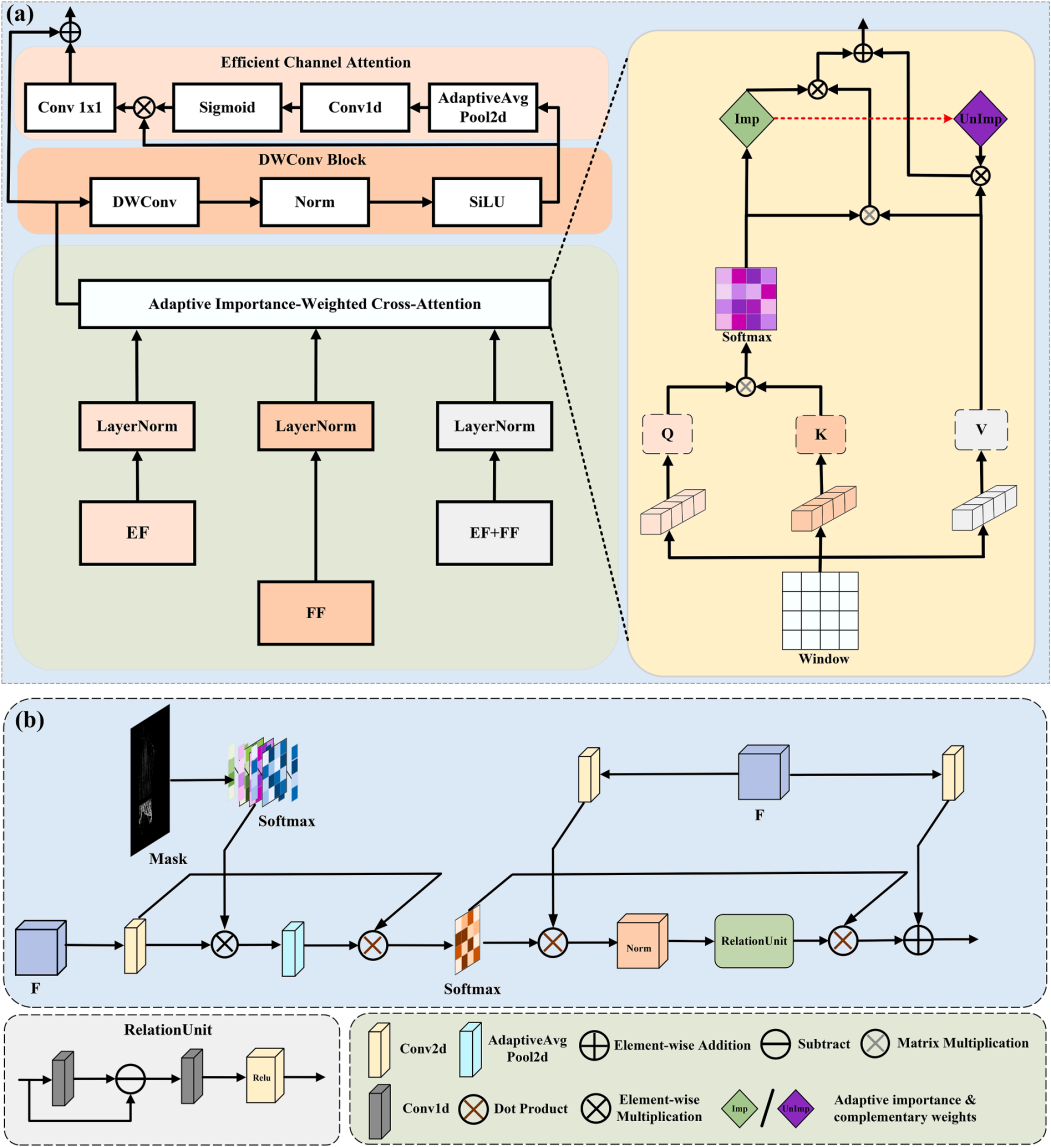
Structures of the Edge-Aware Attention Fusion (EAAF) module and the Anchor Relation Enhancement Module (AREM). (a) The EAAF module fuses boundary and semantic features within local windows through Q, K, and V projections. The derived importance factors balance the attention-enhanced features and the original value features. The output is further refined by depthwise convolution, Efficient Channel Attention (ECA), and a residual connection. (b) The AREM projects the input feature map into a low-dimensional state space. The attention prior mask guides the generation of anchor features by adaptive pooling. These anchors are used to construct an anchor-pixel relation matrix. The RelationUnit models feature interactions, and the output is restored by convolution and fused with the input through a residual connection.

In the decoder, we propose the Edge-aware Relational Fusion Head (ERFHead) (Fig. 2d). ERFHead is designed to fuse edge information with semantic context. The decoding process includes three main steps. First, Edge-Aware Attention Fusion (EAAF) is used to combine shallow semantic features with boundary features (Fig. 3a). This step enhances boundary representation in shallow feature maps. Second, multi-scale Transformer features are used to generate an auxiliary prior mask (Fig. 2c). This mask provides guidance for the Anchor Relation Enhancement Module (AREM) (Fig. 3b). AREM performs relational propagation across feature maps. Third, multi-scale linear fusion is used to generate the final segmentation prediction.

### 2.3 Encoder of ELMERF

#### 2.3.1 Boundary feature extraction branch

The EL-CNN branch (Fig. 2a) takes grayscale images $I_{\text{gray}}\in R^{B\times 1\times H\times W}$ as input, where B is the number of images. It uses the Laplacian of Gaussian (LoG) operator to extract boundary information. The input RGB image is first converted into $I_{\text{gray}}$. Multi-scale LoG kernels $\kappa_{\sigma_{i}}\left(x,y\right)$ are then applied to compute edge responses (Eq. (3)). Here, (x,y) denotes the image coordinates, and $\sigma_{i}$ controls the Gaussian smoothing scale. For each scale, $I_{\text{gray}}$ is convolved with $\kappa_{\sigma_{i}}\left(x,y\right)$. The absolute responses are aggregated by pixel-wise max pooling across all scales to obtain an intermediate edge-response map t (Eq. (4)). The min-max normalization function Norm(·) is defined in Eq. (5). The final multi-scale edge-response map $E_{\mathrm{LoG}}$ is obtained by applying this normalization to t (Eq. (6)). A small constant ε is used to avoid division by zero.

(3) $$\kappa_{\sigma_{i}}\left(x,y\right)=\frac{x^{2}+y^{2}-2{\sigma_{i}}^{2}}{{\sigma_{i}}^{4}}\exp\left(-\frac{x^{2}+y^{2}}{2{\sigma_{i}}^{2}}\right)$$

(4) $$t=\max_{i}\left|\kappa_{\sigma_{i}}*I_{\text{gray}}\right|$$

(5) $$\text{Norm}\left(x\right)=\frac{x-\min\left(x\right)}{\max\left(x\right)-\min\left(x\right)+\varepsilon }$$

(6) $$E_{\mathrm{LoG}}=\text{Norm}\left(t\right)$$

Since LoG responses may contain spurious boundaries in texture-rich regions, a DenoiseBlock $f_{denoise}\left(\cdot \right)$ is applied after LoG filtering. This block consists of two 3×3 convolutions with GroupNorm and GELU activation, followed by an SE block [26]. It produces the denoised feature representation $E_{\text{Denoise}}\in R^{B\times C_{D\_\text{out}}\times H\times W}$ (Eq. (7)). The denoised feature map is then passed through a StemBlock. This operation generates a 1/4-resolution feature map $E_{s4}\in R^{B\times C_{S\_\text{out}}\times \frac{H}{4}\times \frac{W}{4}}$ (Eq. (8)). Two progressive encoding stages, Stage-0 and Stage-1, are further used for boundary feature extraction. Each stage is composed of stacked ConvBlocks, including depthwise separable convolution, layer normalization, pointwise FFN, residual connection, and DropPath. Stage-0 operates on $E_{4}$, and its transformation is defined in Eq. (9). Its output is recalibrated by an SE module to obtain $E_{4}\in R^{B\times C_{4}\times \frac{H}{4}\times \frac{W}{4}}$ (Eq. (10)). Stage-1 further processes $E_{4}$ using deeper ConvBlocks. After SE recalibration, it outputs $E_{8}\in R^{B\times C_{8}\times \frac{H}{8}\times \frac{W}{8}}$ (Eq. (11)).

Finally, EL-CNN outputs two edge-sensitive feature maps at different scales, {$E_{4}$, $E_{8}$}, where $E_{i}\in R^{B\times C_{i}\times \frac{H}{i}\times \frac{W}{i}}$. These boundary features are aligned with the multi-scale semantic features in the decoder. They provide structural cues for subsequent feature fusion.

(7) $$E_{\text{Denoise}}=\text{SE}\;\left[f_{denoise}\right]$$

(8) $$E_{s4}=\text{Stem}\left(E_{\text{Denoise}}\right)$$

(9) $$X_{\text{out}}=\text{FFN}\left(\mathrm{LN}\left(\text{DWConv}\left(E_{s4}\right)\right)\right)$$

(10) $$E_{4}={\text{SE}}_{0}\left[E_{s4}+\text{DropPath}\left(\gamma \cdot X_{\text{out}}\right)\right]$$

(11) $$E_{8}={\text{SE}}_{1}\left(\text{ConvBlock}\left(E_{4}^{↓}\right)\right)$$

#### 2.3.2 Feature extraction branch

In EL-MViT, the Transformer-based feature extraction branch uses the Mix Transformer (MiT) backbone [25] (Fig. 2b). MiT takes RGB images $I\in R^{B\times 3\times H\times W}$ as input. It generates multi-scale feature maps F = {$M_{4}$, $M_{8}$, $M_{16}$, $M_{32}$}, where $M_{i}\in R^{B\times C_{i}\times \frac{H}{i}\times \frac{W}{i}}$. Each Transformer stage uses multi-head self-attention for feature modeling (Eq. (12)). To reduce computational complexity, MiT downsamples K and V during attention computation. Their sequence length is reduced from N to N/R, where R is the reduction ratio. This reduces the computational complexity from $O\left(N^{2}\right)$ to $O\left(N^{2}/R\right)$. A 3×3 depthwise convolution is also introduced into the Mix-FFN module. This operation provides local positional information and improves spatial feature representation.

(12) $$\text{Attn}\left(Q,K,V\right)=\text{Softmax}\left(\frac{QK^{⊤}}{\sqrt{d_{\text{head}}}}\right)V$$

MiT provides several configurations from B0 to B5. These configurations differ in channel dimension, network depth, number of attention heads, and downsampling ratio. In this study, we used the pretrained MiT-B1 configuration as the semantic backbone, considering its balance between segmentation accuracy and parameter size.

### 2.4 ERFHead

The ERFHead (Fig. 2d) is a feature-fusion decoder that explicitly incorporates boundary information. Its inputs include four levels of semantic feature maps {$M_{4}$, $M_{8}$, $M_{16}$, $M_{32}$} generated by the MiT and two levels of edge feature maps {$E_{4}$, $E_{8}$} extracted by the EL-CNN. The semantic feature maps are projected to a unified dimension d by MLPs. The boundary feature maps are aligned by 1×1 convolutions. This produces {$S_{4}$, $S_{8}$, $S_{16}$, $S_{32}$, $R_{4}$, $R_{8}$}, where $S_{i},R_{i}\in R^{B\times d\times \frac{H}{i}\times \frac{W}{i}}$.To provide intermediate supervision and a guidance prior (Fig. 2c), $S_{32}$ is first refined by a Convolutional Block Attention Module (CBAM) [27]. Then $S_{8}$, $S_{16}$, and $S_{32}$ are upsampled to the spatial resolution of $S_{4}$. These feature maps are concatenated along the channel dimension. A 1×1 convolution is then applied to obtain a temporary fused feature map. The classification head generates auxiliary logits from this feature map. The logits are passed through Softmax. The foreground channels are then summed to obtain a single-channel mask. This mask is used as the guidance signal for the feature maps at each scale in the subsequent AREM.

At the shallow resolutions, $R_{4}$ is fused with $S_{4}$, and $R_{8}$ is fused with $S_{8}$. Fusion is performed by channel concatenation, followed by a 1×1 convolution and CBAM. This produces {$C_{4}$, $C_{8}$}, where $C_{i}\in R^{B\times d\times \frac{H}{i}\times \frac{W}{i}}$. The fused feature maps are then passed through two EAAF modules, producing the boundary-enhanced shallow features {$F_{4}$, $F_{8}$}. Then AREM modules are applied to {$F_{4}$, $F_{8},S_{16}$, $S_{32}$}. Guided by the mask generated from the auxiliary logits, each module performs anchor aggregation and global relation modeling. The resulting feature maps are bilinearly upsampled to the spatial size of $F_{4}$. Finally, the four enhanced semantic feature maps are concatenated along the channel dimension, followed by a 1×1 convolution, Dropout, and a classification head to generate the final logits.

#### 2.4.1 Edge-aware attention fusion

The Edge-Aware Attention Fusion (EAAF) module uses boundary priors to refine semantic features (Fig. 3a). The boundary features $X_{\text{EF}}\in R^{B\times C\times H\times W}$ and the semantic features $X_{\text{FF}}\in R^{B\times C\times H\times W}$ are first normalized. They are then fused to obtain $X_{\text{fused}}\in R^{B\times C\times H\times W}$ (Eq. (13)). The fused feature map is partitioned into non-overlapping local windows. In each window, Q, K, and V are generated by linear projections (Eq. (14)). $W_{q},W_{k}{,W}_{v}$ are the corresponding projection matrices. The attention weights A are computed from Q and K with $d_{k}$ as the scaling factor (Eq. (15)). The weighted features $X_{\text{attn}}$ are obtained by applying A to V (Eq. (16)).

(13) $$X_{\text{fused}}=\text{Norm}\left(X_{\text{EF}}+X_{\text{FF}}\right)$$

(14) $$Q=W_{q}X_{\text{EF}},K=W_{k}X_{\text{FF}},V=W_{v}X_{\text{fused}}$$

(15) $$A=\text{Softmax}\left(\frac{QK^{⊤}}{\sqrt{d_{k}}}\right)$$

(16) $$X_{\text{attn}}=A\cdot V$$

An importance weighting mechanism is used in EAAF to adjust the contribution of different local windows. This mechanism is based on the attention matrix generated within each window. For the local attention matrix $A\in R^{B\times h\times N\times N}$, h denotes the number of heads, and N denotes the number of pixels within a window. The absolute value of A is first calculated. The mean value across the spatial and head dimensions is then used to obtain the window-level importance score Imp (Eq. (17)). The importance score Imp is normalized to the range [0,1] (Eq. (18)). A small constant ϵ is used to avoid division by zero. The normalized Imp weights the attention-enhanced feature map $X_{\text{attn}}$. Its complementary weight UnImp preserves information from the original value feature map V (Eq. (19)). The final fused output $X_{\text{out}}$ is obtained as defined in Eq. (20). This design balances attention-enhanced features and original local features during fusion.

(17) $$\text{Imp}=\frac{1}{hN^{2}}\sum_{i=1}^{h}\sum_{j=1}^{N}\sum_{k=1}^{N}\left|A_{i,j,k}\right|$$

(18) $$\text{Imp}=\frac{\text{Imp}}{\max\left(\text{Imp}\right)+\epsilon }$$

(19) UnImp=(1−Imp)

(20) $$X_{\text{out}}=\text{Imp}\cdot X_{\text{attn}}+\text{UnImp}\cdot V$$

After obtaining the attention-enhanced features, EAAF applies convolutional enhancement and ECA [28] to capture local spatial context and recalibrate channel responses (Eq. (21)). A linear projection and a residual connection are then used to stabilize feature transformation. This process further refines boundary-sensitive features at shallow stages.

(21) $$Y=\text{Proj}\left(\text{ECA}\left(\text{Conv}\left(X_{\text{out}}\right)\right)\right)+X_{\text{fused}}$$

#### 2.4.2 Anchor Relation Enhancement Module

The Anchor Relation Enhancement Module (AREM) models spatial dependencies through anchor-guided relation propagation (Fig. 3b). It takes the feature maps {$F_{1}$, $F_{2}$, ${\;S}_{3}$, $S_{4}$} from ERFHead as input. It also uses the attention prior mask $M\in R^{B\times 1\times H\times W}$ generated by deep supervision (Fig. 2c). AREM first applies two 1×1 convolutions to generate the state features $X_{s}\in R^{B\times C^{\prime }\times L}$ and the projection features $X_{p}\in R^{B\times C^{\prime }\times H\times W}$, where L = H·W. The attention prior mask is multiplied with $X_{p}$ to obtain $X_{\text{mask}}$ (Eq. (22)). Adaptive pooling is then used to compress $X_{\text{mask}}$ into a fixed number of anchor features $X_{\text{anchor}}\in R^{B\times C^{\prime }\times N}$, where N is the number of anchors. The anchor features are used to construct the relation matrix R $\in R^{B\times N\times L}$ (Eq. (23)). Here, $X_{p}^{\text{flat}}\in R^{B\times C^{\prime }\times L}$ denotes the unfolded projection features. The state features are propagated from the pixel space to the anchor space through the relation matrix to obtain $X_{\text{rel}}$ (Eq. (24)). A RelationUnit (Fig. 3b) is then applied to model interactions among the anchor-level features (Eq. (25)). The enhanced anchor features are decoded back to the pixel domain using the relation matrix, restored to the original channel dimension by a convolution, and finally fused with the input feature X through a residual connection (Eq. (26)).

(22) $$X_{\text{mask}}=X_{p}⊙M$$

(23) $$R={\text{Softmax}}_{\text{anchors}}\left(X_{\text{anchor}}^{⊤}\cdot X_{p}^{\text{flat}}\right)$$

(24) $$X_{\text{rel}}={\frac{1}{L}X}_{s}\cdot R^{⊤}$$

(25) $$X_{\text{RU}}=\text{RelationUnit}\left(X_{\text{rel}}\right)$$

(26) $$X_{\text{out}}=X+\text{Conv}\left(\text{Reshape}\left(X_{\text{RU}}\cdot R\right)\right)$$

AREM uses the attention prior mask to generate anchor representations from the input features. It then constructs an anchor-pixel relation matrix and performs feature interaction through the RelationUnit. The enhanced features are projected back to the pixel domain and fused with the input through a residual connection.

### 2.5 Loss function

During training, ELMERF uses a primary supervision branch and a deep supervision branch. The primary branch generates the final segmentation output. The deep supervision branch provides auxiliary supervision for intermediate features. This design helps constrain mid-level representations during training. Let the number of classes be K. For a given pixel p, the logits from the primary branch are denoted as $z_{p}\in R^{K}$. The logits from the auxiliary branch are denoted as ${\tilde{z}}_{p}\in R^{K}$. The ground-truth label is denoted as $y_{p}\in \left\{0,\ldots ,K-1\right\}$. Cross-entropy loss is calculated for both branches. Before loss calculation, the outputs are aligned with the ground-truth label size. The primary output is upsampled to the label resolution through the MMSegmentation mechanism (Eq. (27)). The auxiliary output is resized by bilinear interpolation in the decoder head (Eq. (28)).

(27) $$L_{\text{main}}=-\frac{1}{\sum_{p}m_{p}}\sum_{p}m_{p}w_{y_{p}}\;\log\frac{e^{z_{p,y_{p}}}}{\sum_{c=0}^{K-1}e^{z_{p,c}}}$$

(28) $$L_{\text{aux}}=-\frac{1}{\sum_{p}m_{p}}\sum_{p}m_{p}w_{y_{p}}\;\log\frac{e^{{\tilde{z}}_{p,y_{p}}^{↑}}}{\sum_{c=0}^{K-1}e^{{\tilde{z}}_{p,c}^{↑}}}$$

The loss function uses a class-weight vector $w=\left(w_{0},\ldots ,w_{K-1}\right)$. This vector is shared by the primary and auxiliary supervision branches. Pixels labeled as ignore_index are excluded from loss computation. To address the imbalance between abundant background pixels and sparse foreground pixels, we adopted the weighted loss formulation used in ENet [29]. The class weight is calculated as shown in Eq. (29).

(29) $$W_{K}=\frac{1}{\ln\left(c+P_{K}\right)}$$

Here, $P_{K}$ denotes the ratio of pixels belonging to class K to the total number of pixels. The constant c is an additional hyperparameter, and it was set to 1.06 in our implementation. This formulation keeps the class weights within a stable range when the proportion of a foreground class is very low. The total loss is calculated as a linear combination of the main loss and the auxiliary supervision loss. Let $\alpha_{1}$ and $\alpha_{2}$ denote the weighting factors for the primary and auxiliary branches, respectively. In this study, $\alpha_{1}$ and $\alpha_{2}$ were set to 1.0 and 0.4, respectively, as defined in Eq. (30).

(30) $$L=\alpha_{1}\cdot L_{\text{main}}+\alpha_{2}\cdot L_{\text{aux}}$$

## 3 Experiments

### 3.1 RicePhenoSeg

The RicePhenoSeg (RPS) software developed in this study was implemented using Qt [30] and OpenCV [31] (Fig. 1c). RPS provides an interactive interface for image segmentation, region correction, area measurement, mask export, and trait extraction. In this study, it was used as a standardized auxiliary tool for constructing rice seedling segmentation masks and extracting image-derived phenotypic traits.

During image processing, RPS first applied Otsu thresholding [32] to generate an initial segmentation result. The threshold could then be visually adjusted according to image quality. This step was used to handle illumination variation, complex shoot edges, overlapping shoots, and intertwined roots. After threshold segmentation, morphological operations, connected-component analysis, noise removal, and geometric filtering were applied to remove pseudo-targets and connect fragmented target regions. A red reference marker was used for area calibration. The final outputs included green shoot tissue area, yellow shoot tissue area, root area, semantic masks, and spreadsheet files.

Four semantic categories were defined for annotation: background, green shoot tissues, yellow shoot tissues, and roots. Because green and yellow shoot tissues often show gradual transitions under salt stress, a unified annotation procedure was used. Specifically, RPS first generated an initial mask by Otsu thresholding, and the threshold was adjusted when necessary. Green shoot regions were further constrained in the HSV color space. On this basis, non-green discolored regions within the shoot were annotated as yellow shoot tissues. All annotations were performed by trained annotators and checked by researchers experienced in rice salt-stress phenotyping.

The RSSD-A dataset contained 501 manually annotated rice seedling images. The annotation masks were generated using the RPS-assisted workflow and had a resolution of 1000 × 1500 pixels. The dataset was divided into training, validation, and testing sets at a ratio of 8:1:1 for supervised model training and performance evaluation (Table 1).

To evaluate the reliability of the RPS-assisted workflow, Adobe Photoshop 2021 was used as a manual reference. Twenty-five representative images were selected from the 501 salt-stress seedling images, covering different levels of visible shoot yellowing. Yellow shoot tissue area, green shoot tissue area, and root area obtained by RPS were compared with the manually refined Photoshop measurements. As shown in Fig. S3, the Pearson correlation coefficients were 0.973, 0.989, and 0.993 for yellow shoot tissues, green shoot tissues, and roots, respectively. These results indicate that RPS provided consistent measurements across representative samples. Therefore, RPS was used to improve the consistency and efficiency of annotation and phenotypic extraction in large-scale rice seedling images.

### 3.2 Network training and testing

The model was trained on the RSSD-A dataset using PyTorch 2.1.0 and MMEngine 0.10.7. During training, we used a standard data augmentation pipeline, including RandomResize, RandomCrop, RandomFlip, and PhotoMetricDistortion. Specifically, images were randomly resized with a scale range of 0.5–2.0 while maintaining the aspect ratio, and then randomly cropped to 512 × 512 pixels as model inputs. Random horizontal flipping was applied with a probability of 0.5, and photometric perturbations were used to adjust brightness and contrast. Images were normalized using mean values of [123.675, 116.28, 103.53] and standard deviations of [58.395, 57.12, 57.375]. The model used EL-MViT as the backbone with MiT-B1 pretrained weights. The backbone contained four stages with output dimensions of 64, 128, 320, and 512, and the DropPath rate was set to 0.1. The EL-CNN branch included two progressive encoding stages for boundary feature refinement, with feature dimensions of 64 and 128. The SE block used a reduction ratio of 16. The model was optimized using AdamW with an initial learning rate of ${6\times 10}^{-5}$ and a weight decay of 0.01. The learning rate of the decoder head, including the feature projection layers, EAAF modules, AREM modules, feature fusion layers, and final prediction layer, was set ten times higher than the base learning rate. The learning rate was first linearly warmed up for 8 epochs and then decayed using the PolyLR strategy with a power of 1.0. The model was trained for 150 epochs, and validation was performed every 3 epochs. All experiments were conducted on a Linux server equipped with an NVIDIA GeForce RTX 3090 GPU with 24 GB memory, dual Intel Xeon Gold 5218R CPUs, and 128 GB RAM. The same computational environment was used for all compared models to ensure fair comparison and reproducibility.

### 3.3 Evaluation criteria

Semantic segmentation performance was evaluated using four standard metrics: Intersection over Union (IoU), mean Intersection over Union (mIoU), Accuracy (Acc), and mean Accuracy (mAcc) (Eqs. (31)–(34)). For the i-th class, $TP_{i}$, $FP_{i}$, and $FN_{i}$ represent true-positive, false-positive, and false-negative pixels, respectively. K represents the total number of classes. IoU quantifies the overlap between the predicted mask and the ground-truth annotation. mIoU is the average IoU across all classes. Acc is calculated as the ratio of correctly classified pixels to all ground-truth pixels in a given class. mAcc is the average Acc across all classes. These metrics were used for model evaluation and comparison.

(31) $$\text{Io}U_{i}=\frac{TP_{i}}{TP_{i}+FP_{i}+FN_{i}}$$

(32) $$\text{mIoU}=\frac{1}{K}\sum_{i=1}^{K}\text{Io}U_{i}$$

(33) $${\text{Acc}}_{i}=\frac{TP_{i}}{TP_{i}+FN_{i}}$$

(34) $$\text{mAcc}=\frac{1}{K}\sum_{i=1}^{K}{\text{Acc}}_{i}$$

### 3.4 Comparison analysis

To evaluate the performance of our model, we compared the ELMERF model with nine state-of-the-art semantic segmentation networks, including SegFormer [25], SegNeXt [33], Mask2Former [34], MambaVision [35], VMamba [36], ConvNeXt [37], PoolFormer [38], SegMAN [39] and OffSeg [40]. These networks were implemented using the PyTorch-based OpenMMLab model library or their official open-source implementations. We reproduced all models based on the configurations and training details provided in the original papers. To ensure a fair and valid comparison, all models were trained and evaluated on the same dataset under identical settings, and their performance was assessed with the same metrics.

### 3.5 Explainability analysis of ELMERF

The interpretability of ELMERF was evaluated using Grad-CAM visualization and ablation analysis. For qualitative analysis, Grad-CAM [41] was applied to the trained segmentation model to visualize regions contributing to model prediction. Heatmaps from the full model and the variants without EL-CNN, EAAF, or AREM were compared. For quantitative analysis, each module was removed separately. The corresponding segmentation metrics were then compared to estimate its contribution to model performance.

### 3.6 Genome-wide association analysis

A total of 7,762,568 high-quality SNPs with a minor allele frequency (MAF) greater than 0.05 and a deletion rate of less than 0.1 were selected from 261 rice cultivars for GWAS. The SNP dataset was obtained from the RiceVarMap database (http://ricevarmap.ncpgr.cn) [42]. The GWAS analyses were conducted using the Efficient Mixed Model Association expedited (EMMAX) method [43], with the parameters emmax -v -d 10 -t new_all_chr.filter -p pheno -k new_all_chr.filter.hIBS.kinf -o result. The kinship (K) matrix was constructed using the emmax-kin program with the parameters emmax-kin -v -h -s -d 10 new_all_chr.filter. The Manhattan plot was generated using the R package 'qqman' along with custom R scripts. The genome-wide suggestive threshold, derived from the original Bonferroni calculation of 1/Me, was set at –log_10_(*p*) = 5.5 for the entire population. And then we screened candidate genes within the 300 kb region upstream and downstream of the significant loci. This approach has been consistently applied in our prior studies and achieved robust and consistent outcomes [44,45].

### 3.7 Statistical analysis

Statistical analyses other than GWAS were performed using Origin 2024 and Python (v3.8). Descriptive statistics were used to summarize phenotypic traits and image-derived measurements. Pearson correlation analysis and ordinary least-squares (OLS) linear regression were used for continuous traits, whereas Spearman rank correlation analysis was used for ordinal or non-normally distributed traits. Pearson's r, Spearman's ρ, R^2^, and corresponding two-sided P values were reported according to the analysis type. For consistency analysis, ELMERF-derived ratio traits were compared with RPS-derived ground-truth measurements using correlation analysis, OLS regression, error metrics, and Bland–Altman analysis. In Bland–Altman analysis, differences were calculated as ELMERF-derived values minus RPS-derived values, and 95% limits of agreement were defined as bias ±1.96 × SD. Statistical significance was set at P < 0.05 unless otherwise stated.

## 4 Results

### 4.1 ELMERF outperforms most other SOTA networks

**Fig. 4:**
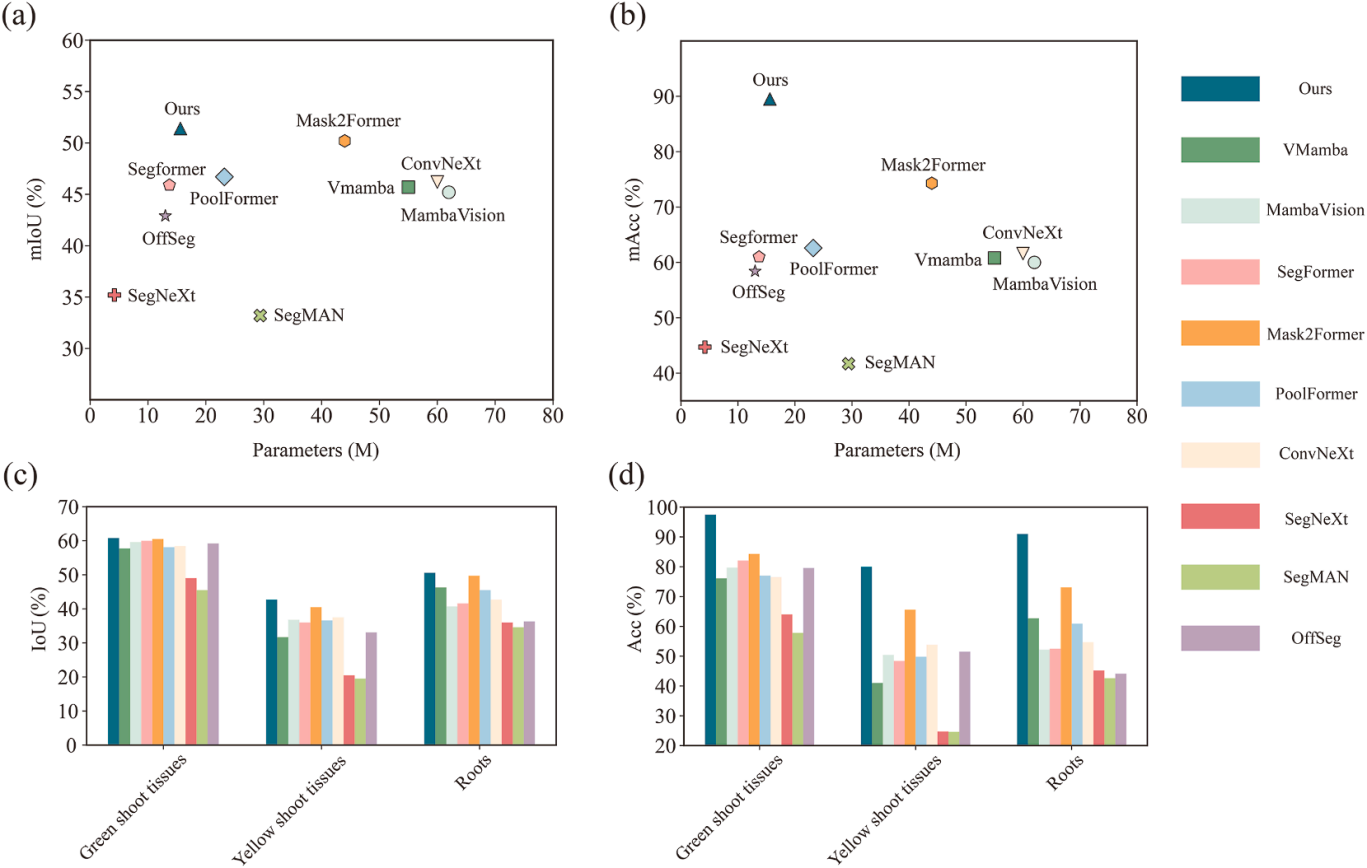
Performance indicators of various segmentation models. (a) Model parameters vs mIoU. (b) Model parameters vs mAcc. (c) IoU performance of each model in three categories (green shoot tissues, yellow shoot tissues, roots). (d) Accuracy (Acc) of each model on the three categories.

**Fig. 5:**
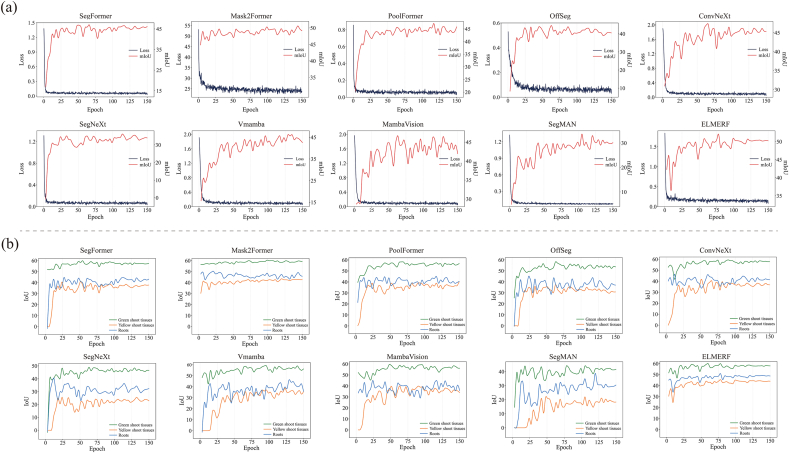
Training dynamics of different segmentation models. (a) Training loss and validation mIoU evolution for all models. (Ours, SegFormer, Mask2Former, PoolFormer, OffSeg, ConvNeXt, SegNeXt, VMamba, MambaVision and SegMAN). (b) Class-level IoU evolution on three categories (green shoot tissues, yellow shoot tissues, roots).

ELMERF achieved a favorable balance between segmentation performance and model complexity (Fig. 4a and b). It contained 15.6 million parameters. Despite this relatively small model size, ELMERF obtained higher mIoU and mAcc than the other compared models (training processes are detailed in Fig. 5), including several models with substantially larger parameter sizes. On the test set, ELMERF achieved an mIoU of 51.4% and an mAcc of 89.5% (Table 2). For the three foreground categories, the IoU values of green shoot tissues, yellow shoot tissues, and roots were 60.8%, 42.7%, and 50.6%, respectively, and the corresponding Acc values were 97.5%, 80.0%, and 91.0%. Compared with nine representative deep learning models, ELMERF exceeded Mask2Former [34], PoolFormer [38], and ConvNeXt [37] by 2.4%, 10.1%, and 11.3% in mIoU, and by 20.5%, 43.0%, and 45.3% in mAcc, respectively. Its mIoU and mAcc were also 18.3% and 53.4% higher than the average values of all nine models.

**Table 2:** Segmentation performance of different models.

Method	Backbone	Decoder	mAcc	mIoU	Parameters
MambaVision	MambaVision-T-1K	UPerHead	60.8%	45.7%	55.0M
OffSeg	OffSeg-B	OffSegHead	58.4%	42.9%	13.0M
SegMAN	SegMAN-S	SegMANDecoder	41.7%	33.2%	29.4M
VMamba	VMamba-T	UPerHead	60.0%	45.2%	62.0M
SegNeXt	MSCAN_T	LightHamHead	44.7%	35.2%	**4.2M**
Mask2Former	R-50	Mask2FormerHead	74.3%	50.2%	44.0M
ConvNeXt	ConvNeXt-T	UPerHead	61.6%	46.2%	60.0M
PoolFormer	PoolFormer_s24	FPNHead	62.6%	46.7%	23.2M
SegFormer	MiT-B1	SegFormerHead	61.0%	45.9%	13.7M
Ours	MiT-B1	ERFHead	**89.5%**	**51.4%**	15.6M

Green and yellow shoot tissues are key phenotypic indicators for evaluating rice salt-tolerance. ELMERF showed clear advantages in key foreground-class segmentation (Fig. 4c and d). For green shoot tissues, ELMERF achieved an IoU 0.5% higher than the second-best model and 7.8% higher than the average. Its Acc was 15.7% higher than the second-best model and 29.7% higher than the average. For yellow shoot tissues, ELMERF achieved an IoU 5.4% higher than the second-best model and 31.4% higher than the average. Its Acc was 22.0% higher than the second-best model and 75.8% higher than the average. For roots, ELMERF achieved an IoU 1.8% higher than the second-best model and 21.9% higher than the average. Its Acc was 24.5% higher than the second-best model and 67.9% higher than the average.

**Fig. 6:**
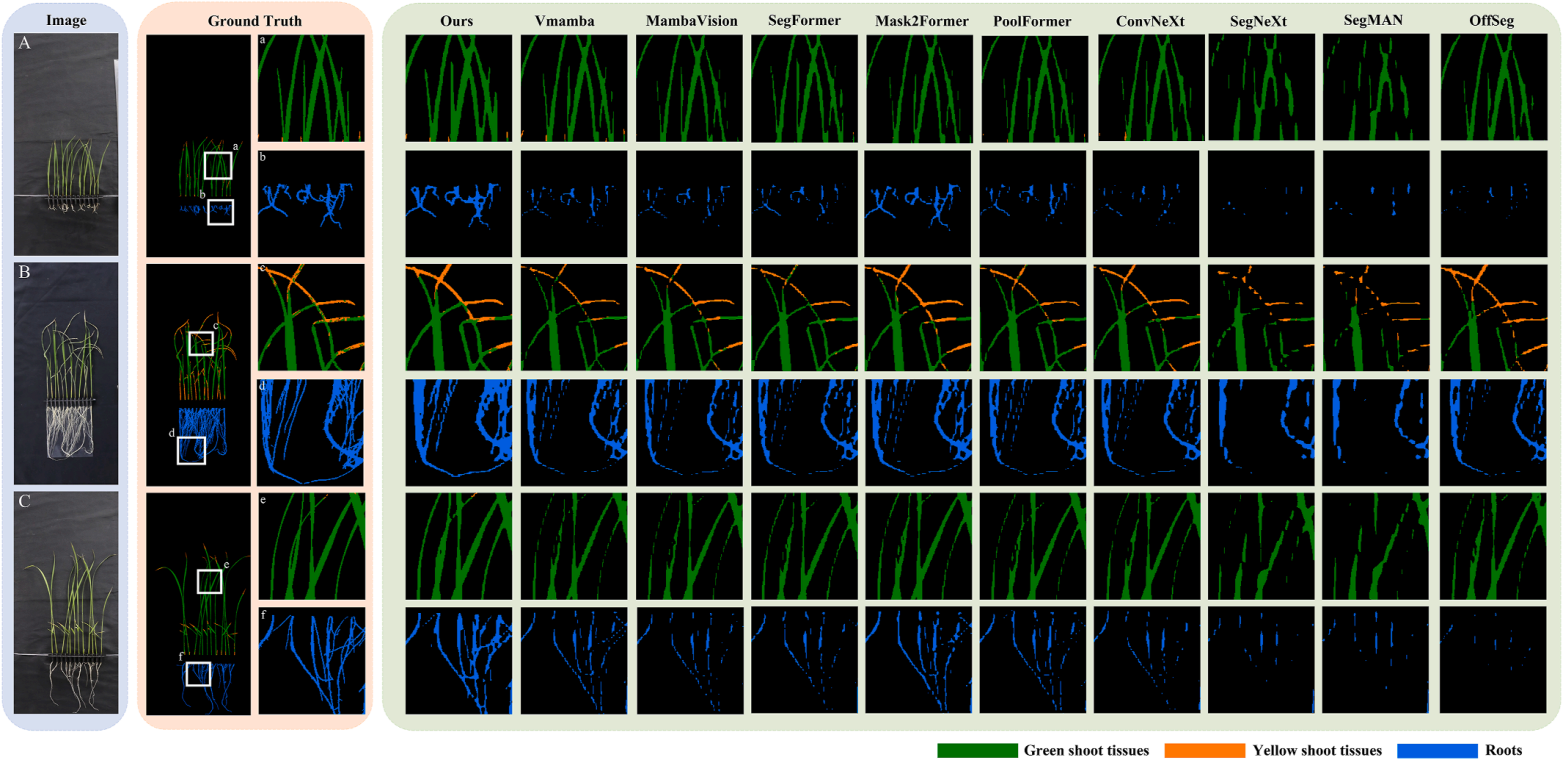
Comparison of segmentation results between different models and ground-truth masks. Examples showing the performance of the models (Ours, VMamba, MambaVision, SegFormer, Mask2Former, PoolFormer, ConvNeXt, SegNeXt, SegMAN and OffSeg).

The RSSD-A is challenging for semantic segmentation because of severe foreground–background imbalance and complex seedling structures. Weakly yellowing regions are often similar to normal green shoot tissues in color and texture, while slender shoots, overlapping organs, and intertwined roots further increase the difficulty of fine foreground segmentation. Despite these challenges, ELMERF produced more complete and coherent segmentation results than the compared models (Fig. 6). This indicates that ELMERF can better preserve key foreground structures and reduce category confusion in complex rice seedling images.

### 4.2 Quantitative characteristics of image-derived phenotypes and comparison with conventional indices

**Fig. 7:**
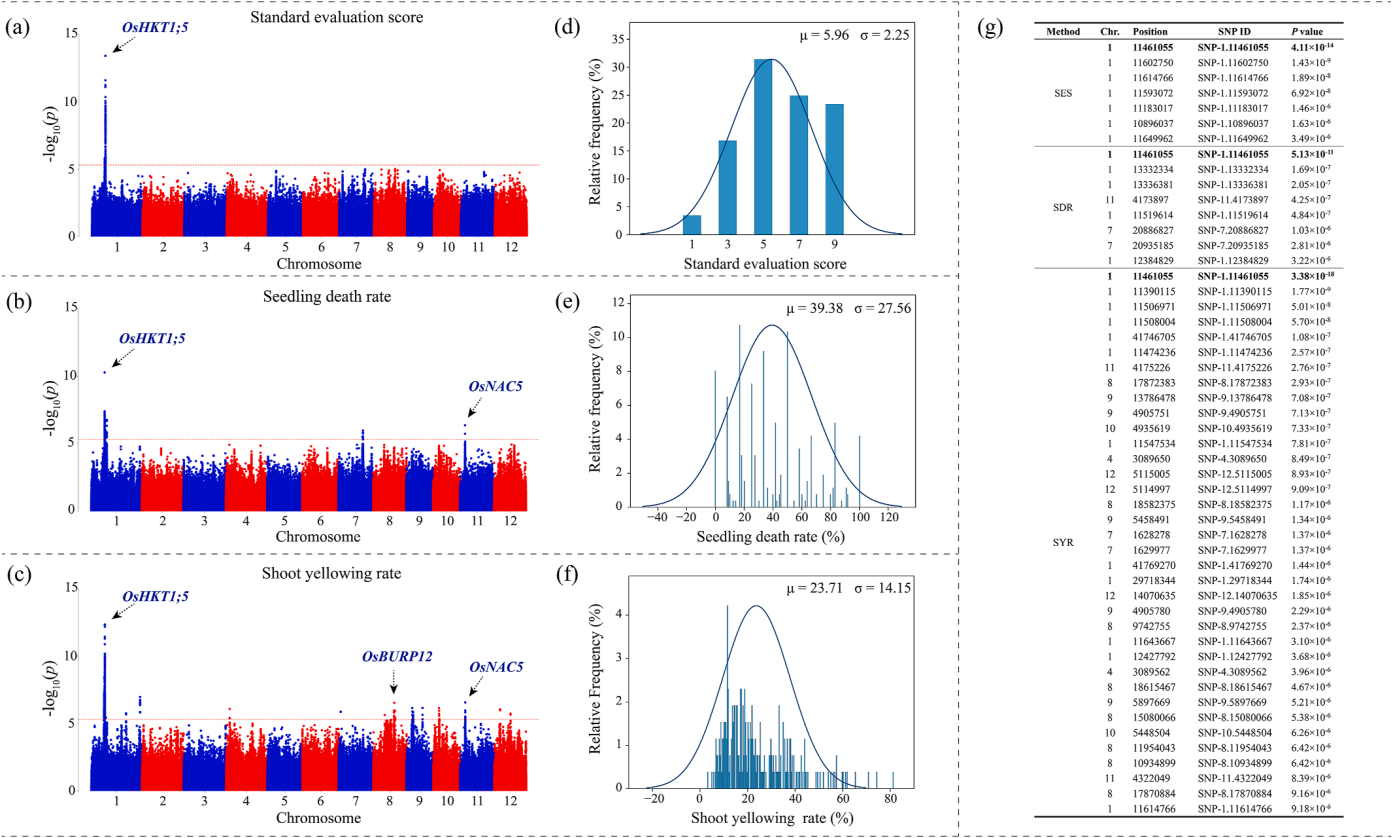
Comparison of phenotypic distributions and GWAS results based on shoot yellowing rate (SYR), seedling death rate (SDR), and standard evaluation score (SES). (a-c) Genome-wide association analysis (GWAS) results for the three phenotypes. The red dashed line indicates the significance threshold, –log_10_(*p*) = 5.5. (d-f) Histograms showing the phenotypic frequency distributions of the three traits. The SYR phenotype exhibits a more continuous distribution pattern and higher resolution. (g) Significant SNPs detected by SES-, SDR-, and SYR-based GWAS. Among the three traits, SYR identified the largest number of significantly associated SNPs and showed the highest levels of statistical significance.

Using the segmentation outputs generated by ELMERF, we calculated shoot yellowing rate as an image-derived trait for visible salt-induced shoot injury. Shoot yellowing rate was defined as the proportion of yellow shoot area relative to the total shoot area. For comparison, standard evaluation score was manually recorded for the same population. Seedling death rate was also measured to describe severe early seedling damage. To show the phenotypic variation captured by shoot yellowing rate, ten representative genotypes were selected and arranged in descending order of seedling death rate (Fig. S4). Linear regression showed that shoot yellowing rate was positively associated with seedling death rate (R^2^ = 0.617). This relationship was stronger than that between standard evaluation score and seedling death rate (R^2^ = 0.511) (Fig. S5). The frequency distributions further showed that shoot yellowing rate had a smoother and more continuous pattern than standard evaluation score and seedling death rate (Fig. 7d–f). These results indicate that shoot yellowing rate provides a quantitative and continuous image-derived phenotype for quantifying visible salt injury at the seedling stage. Therefore, shoot yellowing rate was used as the primary phenotype for downstream GWAS.

### 4.3 Consistency between ELMERF-derived ratio traits and RPS ground truth

To evaluate the reliability of segmentation-derived traits for downstream phenotypic analysis, we further compared two ratio-based traits derived from ground-truth masks and model predictions in the 261 images. For ease of presentation, the shoot yellowing rate (SYR) is referred to as the yellow shoot tissue ratio in this section.

The results showed strong correlations between predicted and ground-truth values for the yellow shoot tissue ratio (Pearson r = 0.9661, Spearman ρ = 0.9564, R^2^ = 0.9334). Similar results were observed for the total shoot tissue ratio (Pearson r = 0.9675, Spearman ρ = 0.9679, R^2^ = 0.9361) (Fig. S6a and S6b). These results indicate that the model could largely preserve the relative trends and ranking relationships of phenotypic differences among samples. We further used Bland–Altman analysis to assess the agreement between model predictions and manual annotations. The results showed only a small systematic bias for the total shoot tissue ratio. For the yellow shoot tissue ratio, although a certain underestimation trend was observed, most samples were still within the 95% limits of agreement (Fig. S6c and S6d). These results suggest that, although yellow shoot tissue ratio was slightly underestimated, the prediction errors were relatively consistent across most samples. Therefore, the shoot yellowing rate index can still provide useful information for population-level relative phenotypic comparison.

### 4.4 Genome-wide association analysis based on deep-learning-derived traits

To assess the utility of image-derived phenotypes in genetic mapping, GWAS was conducted separately using standard evaluation score, shoot yellowing rate and seedling death rate as inputs. For all three phenotypes, the same significant SNPs were detected on chromosome 1 at approximately 11.46 Mb (Fig. 7a–c), indicating that the population material and phenotypic data in this study can effectively reflect the real genetic variation in rice seedlings under salt stress. The corresponding Q-Q plots indicate that the test statistics in this study were well-calibrated and that true association signals were present (Fig. S7). This interval contained the known salt tolerance-related gene *OsHKT1;5*, which encodes a sodium transport protein with a key role in maintaining Na^+^ exclusion and ion homeostasis [46,47].

Compared with seedling death rate and standard evaluation score, the shoot yellowing rate-based GWAS detected a stronger lead signal (P = 3.38 × 10^−18^) and identified 36 significant SNPs across chromosomes 1, 4, 7–12 (Fig. 7c and g). Several previously reported stress-responsive genes were recovered from these SNPs, including *OsNAC5* (Chr11, LOC_Os11g08210), which participates in ABA and salt-stress signaling, and *OsBURP12* (Chr8, LOC_Os08g29200), which was recently validated as a negative regulator of salt sensitivity (Table S2). In addition, we identified putative priority candidate genes located near significant SNPs that may be associated with seedling salt-stress responses (Table S3). These findings show that shoot yellowing rate can recover a known salt-tolerance signal and provide additional candidate loci for further analysis. This supports the use of image-derived continuous phenotypes in seedling-stage GWAS.

## 5 Discussion

### 5.1 Reliability of phenotypic extraction under controlled hydroponic conditions

Reliable salt-tolerance assessment depends strongly on the stability of the cultivation environment. Salt response in rice is influenced by both genotype and environmental conditions. Therefore, reducing environmental variation is necessary for obtaining reproducible seedling phenotypes. In this study, we established a controlled hydroponic system for image-based salt-stress phenotyping. The system integrated root-zone management, uniform lighting, and airflow regulation.

Root-zone conditions are a major source of variation in hydroponic experiments. Static hydroponics may lead to the accumulation of root exudates and allelopathic compounds. These factors can introduce additional chemical stress that is independent of salinity [48]. To reduce this effect, physical root isolation and nutrient-solution circulation were introduced into the system (data not shown; Fig. 1a.Ⅱ.Ⅲ). These designs helped reduce phenotypic bias caused by root interactions and nutrient-gradient changes. Light is a fundamental environmental factor driving photosynthesis [49]. Excessive, insufficient, or uneven light may induce chlorosis and morphological variation. This can reduce the precision of image-based phenotypic analysis. To minimize this source of variation, a three-sided illumination design was used. This design maintained light intensity variation within 5% across the hydroponic area (Fig. S1). It also helped reduce local lighting bias and non-salt-induced leaf yellowing. Ventilation was further optimized to stabilize the local microclimate. In enclosed hydroponic systems, airflow regulation helps maintain seedling physiology and canopy gas exchange [50]. Therefore, a horizontal fan-induced airflow system was integrated into the platform (Fig. 1a). This design improved air circulation and reduced microenvironmental heterogeneity among seedlings.

Two independent repeatability experiments showed that the platform produced stable phenotypic responses across genotypes (Fig. S2). In particular, the consistent shoot yellowing rate values observed in the validation experiments indicate that the controlled conditions minimized G×E fluctuations that often confound early-stage salt-tolerance evaluation. The uniformity of phenotypic responses across replications supports the suitability of this system for high-throughput image-derived phenotyping and downstream genetic analysis.

### 5.2 Network interpretability and ablation experiments

To assess the contribution of each functional component, a systematic ablation study was conducted. The results demonstrate that the EL-CNN, the EAAF, and the AREM all play essential roles in improving the overall performance of the model. The complete ELMERF model achieves the highest segmentation accuracy, with an mIoU of 51.4%. When the EL-CNN, EAAF, and AREM were removed individually, performance decreased by 2.2%, 3.0%, and 10.5%, respectively (Table 3). These findings confirm that all three modules are indispensable for effective multi-scale feature extraction and boundary representation.

**Table 3:** The ablation analysis results of ELMERF.

Base-Model	EL-CNN	EAAF	AREM	mIoU
**✓**		**✓**	**✓**	50.3%
**✓**	**✓**		**✓**	49.9%
**✓**	**✓**	**✓**		46.5%
**✓**	**✓**	**✓**	**✓**	**51.4**%

**Fig. 8:**
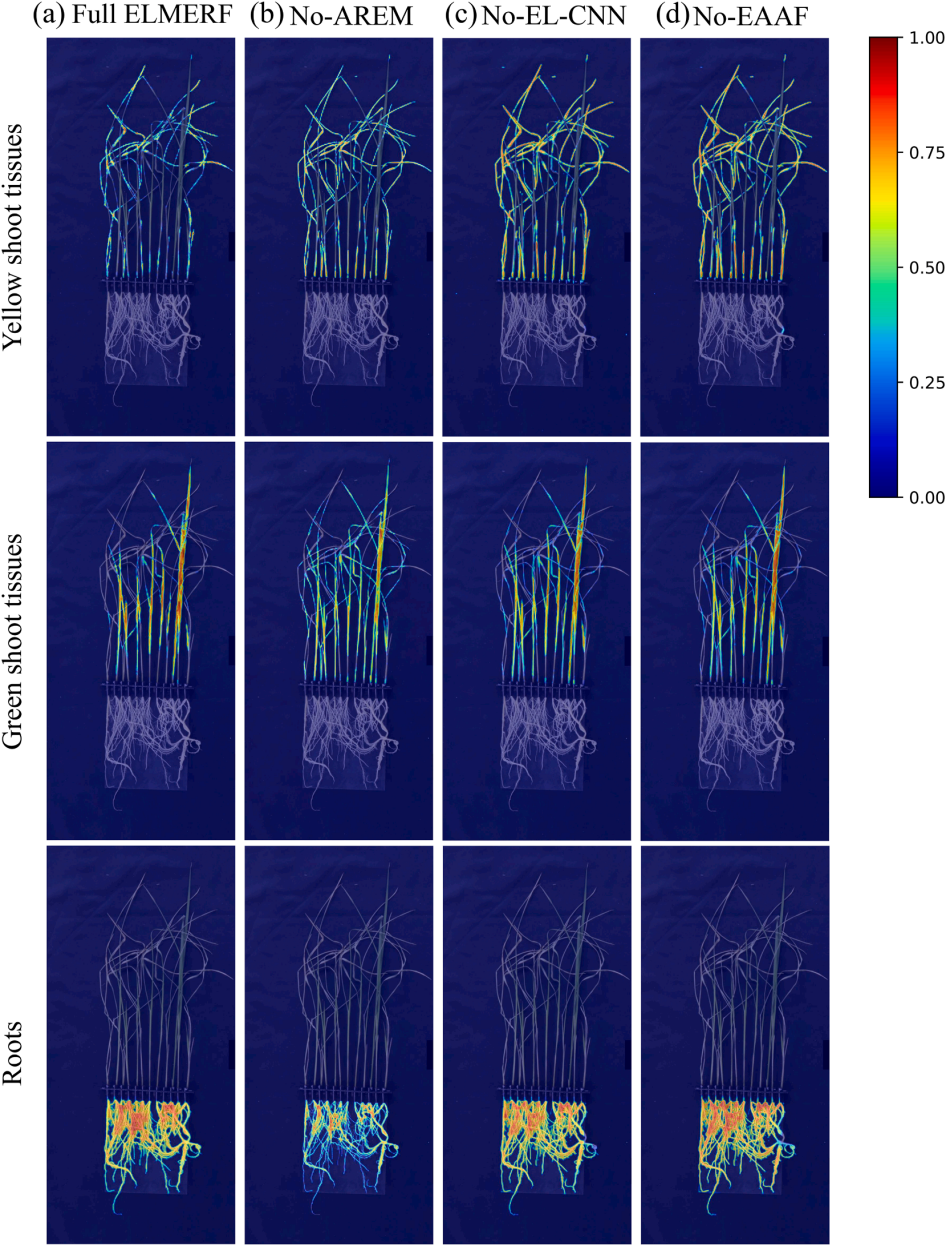
Feature-map visualization for ablation analysis. (a) Full ELMERF model. (b) ELMERF without the Anchor Relation Enhancement Module (No-AREM). (c) ELMERF without the boundary feature extraction branch (No-EL-CNN). (d) ELMERF without the Edge-Aware Attention Fusion module (No-EAAF). Rows show feature-map responses for green shoot tissues, yellow shoot tissues, and roots, respectively. The color bar indicates normalized activation intensity from 0 to 1.

The ablation-based interpretability analysis further shows the contribution of each module (Fig. 8). Removing EL-CNN weakened the response to fine edge structures and increased confusion between yellow and green shoot tissues. Without EAAF, semantic and boundary features were less effectively integrated, leading to blurred object boundaries and more category confusion. Removing AREM reduced attention in complex regions, such as dense root areas. In contrast, the full model produced stronger and more localized activations along shoot edges and root contours. These results indicate that EL-CNN, EAAF, and AREM jointly improve boundary recognition and semantic modeling in complex seedling images.

### 5.3 Segmentation challenges and sources of foreground-class errors

The segmentation of yellow shoot tissues and fine foreground structures remained challenging in RSSD-A. This was mainly related to the pronounced foreground-background imbalance and the intrinsic visual complexity of salt-stressed rice seedlings. Most pixels in the images belonged to the background, whereas the foreground classes, especially yellow shoot tissues and roots, occupied much smaller pixel proportions. This long-tailed distribution limited the number of effective training pixels for minority foreground classes. It also partly explains why yellow shoot tissues and roots showed lower pixel-level IoU and Acc than green shoot tissues. In addition, green and yellow shoot tissues often showed gradual transitions rather than sharp boundaries. Weakly yellowing regions were sometimes similar to normal green tissues in color, brightness, and texture. Slender and overlapping leaves, intertwined roots, and low-contrast root regions further increased the difficulty of accurate pixel assignment (Fig. S8).

Local over-segmentation and false-positive foreground predictions were also observed in some complex samples. This may be related to the class-weighted loss used to reduce the strong foreground-background imbalance. Because the class weights were calculated from the global pixel distribution of the whole dataset, they could not be dynamically adjusted according to the foreground composition of each individual image. When the local proportion of roots or yellow shoot tissues differed from the global average, the model could still overcompensate minority foreground classes. This led to slightly enlarged predicted masks in some samples (Fig. S9). These cases reflect a trade-off between reducing missed detection of minority classes and avoiding false-positive foreground prediction.

Therefore, ELMERF should not be interpreted as providing perfect pixel-level segmentation for every individual sample. Instead, under standardized imaging and processing conditions, the main purpose of the segmentation output in this study was to extract ratio-based phenotypic traits for population-level comparison. The strong consistency between ELMERF-derived traits and RPS-derived ground truth (Fig. S6) supports this purpose. Although local pixel-level errors remained, the model largely preserved the relative trends and ranking relationships among samples. This supports the use of SYR for relative phenotypic comparison and downstream GWAS, while also defining the applicable scope of the method.

### 5.4 Interpretation of image-derived SYR in salt-stress evaluation

Shoot yellowing is an early and visible manifestation of salt-induced shoot injury, whereas seedling death represents a more severe endpoint outcome after stress and recovery. In this study, shoot yellowing rate (SYR) and seedling death rate were therefore used to describe different but related aspects of seedling salt response. Shoot yellowing rate quantified the proportion of yellow shoot tissue immediately after salt treatment, while seedling death rate recorded the proportion of seedlings showing complete loss of greenness in the central leaf after the recovery period. The positive association between shoot yellowing rate and seedling death rate (R^2^ = 0.617) (Fig. S5) indicates that stronger shoot yellowing was generally related to a higher probability of subsequent seedling death. However, this relationship was not one-to-one, suggesting that yellowing and death should not be treated as equivalent phenotypes. In the present annotation scheme, the yellow class represented visibly discolored shoot tissues, mainly reflecting chlorotic or yellowing regions under salt stress. In rice, leaf yellowing is generally associated with chlorophyll degradation and stress-induced deterioration of photosynthetic tissues [51], whereas salt stress can further promote ionic imbalance, osmotic stress, oxidative damage, membrane injury, and senescence-related processes [[52], [53], [54]]. Under severe or prolonged stress, visible injury may further progress toward irreversible tissue damage or necrosis [54]. Therefore, yellow shoot tissue should not be interpreted as dead tissue in all cases. Rather, shoot yellowing rate should be regarded as a standardized image-derived indicator of visible shoot injury under salt stress, not as a direct measurement of tissue death or a mechanistic readout of a single physiological process.

### 5.5 Interpretation of image-derived SYR in genetic mapping

Shoot yellowing rate provides a quantitative proxy for visible shoot-level salt injury (Fig. 7d–f). In GWAS, all three phenotypes detected the major peak on chromosome 1 corresponding to the Saltol region harboring *OsHKT1;5* [46,47]. This shared signal indicates that the population and phenotyping strategy captured a known genetic component of seedling salt response. However, SYR-based GWAS produced a stronger lead signal (P = 3.38 × 10^−18^) and identified more associated SNPs than standard evaluation score or seedling death rate (Fig. 7g). The SYR-based GWAS detected significant signals on chromosomes 11 and 8 that colocalize with reported salt-responsive genes including *OsNAC5* (LOC_Os11g08210) and *OsBURP12* (LOC_Os08g29200). *OsNAC5* is a transcription factor involved in drought, salt, and ABA signaling [55]. *OsBURP12* has been reported as a negative regulator of salt sensitivity in rice [56]. Rather than replacing conventional standard evaluation score or seedling death rate measurements, shoot yellowing rate provides a complementary continuous phenotype that captures visible shoot injury at higher resolution and may increase the sensitivity of GWAS under controlled seedling-stage salt-stress conditions.

Notably, among the significant SNPs detected in this study, we did not observe co-localization with previously reported senescence-regulating genes. This may be because shoot yellowing rate mainly reflects acute salt-induced shoot injury at the seedling stage, and it is different from natural developmental senescence. However, this does not exclude the possibility that salt stress-induced leaf injury and senescence-related pathways may partially overlap at certain downstream stages. In addition, classical physiological indicators were not measured in this study (e.g., Na^+^/K^+^ ratios, chlorophyll content, and antioxidant enzyme activity). The physiological interpretability of shoot yellowing rate remains indirect, and it should be regarded as an image-derived proxy of salt-induced injury. Future studies combining image-derived traits with physiological assays will be needed to further validate the biological basis of shoot yellowing rate.

### 5.6 Limitations and further work

The current workflow was developed and evaluated under a controlled hydroponic system, single-view RGB imaging, a highly uniform background, and a consistent imaging setup. Cross-platform external validation was not included in the present study. A color calibration card was not included during image acquisition, which may limit absolute color standardization across imaging batches, devices, or platforms. Therefore, the present framework should be interpreted as a research-oriented phenotyping pipeline for standardized indoor salt-stress evaluation and population-level genetic analysis. Furthermore, in the present study, foreground-class segmentation remained challenging because of the pronounced class imbalance of the dataset and the intrinsic visual complexity of salt-stressed rice seedling images. Moreover, the current raw dataset should be further expanded in future work to improve model training, evaluation, and generalization.

Although the segmentation framework includes a root category, root traits were not included in the downstream genetic analysis. Quantitative interpretation of root responses would require a dedicated experimental design with appropriate controls and independent validation. Therefore, the present study does not draw conclusions regarding root-based salt-tolerance mechanisms. Nevertheless, the ability of our segmentation system to rapidly quantify root-area traits across diverse genotypes provides a basis for future studies of root responses to salt stress.

The current framework still relies on fully supervised learning with pixel-level annotations generated under a specific experimental setting. Future work could combine this seedling dataset with semi-supervised learning or active-learning strategies to reduce annotation costs and improve model generalization across other plant species and abiotic stress conditions. In addition, integration of multi-temporal image data with time-series modeling approaches could help characterize dynamic salt-stress responses and recovery processes. Future studies could explore its extension to other abiotic stress scenarios, such as drought, cold damage, and evaluate whether similar image-derived traits can provide robust phenotypic inputs under these conditions. In parallel, integration with additional sensing modalities, such as hyperspectral imaging, infrared imaging, or chlorophyll fluorescence imaging, may help link visible injury traits with physiological responses. Multi-angle or three-dimensional imaging could also be explored to extend the current two-dimensional projection-based framework toward more comprehensive seedling phenotyping.

## 6 Conclusions

In conclusion, we established a controlled hydroponic RGB phenotyping framework for rice seedling salt-stress evaluation. By integrating standardized low-cost imaging, ELMERF-based semantic segmentation, and shoot yellowing rate (SYR) extraction, the framework enabled efficient and quantitative assessment of visible shoot injury. Compared with conventional visual scores and endpoint death-rate measurements, SYR improved phenotypic resolution and supported downstream GWAS. The recovery of the known Saltol/OsHKT1; 5 signal and additional candidate loci further supports the value of image-derived traits for rice seedling salt-tolerance analysis. Nevertheless, its transferability to other platforms, environments, stress conditions, and crop species requires further validation.

## Author contributions

Y.L.L. and X.H.M. contributed equally to this work. X.H.M. led the image segmentation algorithm development and contributed to manuscript drafting. Y.L.L. led the experiments (excluding algorithm development), data acquisition and analysis, and contributed to manuscript drafting. Q.Y.X. assisted with data collection and hydroponic system setup. C.X. assisted with data collection. R.X. contributed to study design and provided funding support. H.L. contributed to guiding model optimization and data analysis. X.D.B. supervised the algorithm development and manuscript revision. Z.Y. led the GWAS/genetic mapping analyses and manuscript revision. H.Y.L. designed and supervised the overall study, participated throughout the experiments, revised the manuscript, and provided funding. All authors approved the final manuscript.

## Data availability

The datasets, source code, and other supporting data are openly available on the ELMERF repository (https://github.com/PhenoCodexh/ELMERF).

## Funding

This work was supported by the National Key R&D Program of China (2023YFF1002100), the Nanhai Nova Project in Hainan Province (grant no. NHXXRCXM202308), the National Natural Science Foundation of China (62462027), the Hainan Provincial Natural Science Foundation of China (625MS045) and the Scientific Research Fund Project of Hainan University (KYQD(ZR)-23113).

## Declaration of competing interest

The author is an Editorial Board Member/Editor-in-Chief/Associate Editor/Guest Editor for this journal and was not involved in the editorial review or the decision to publish this article.

The authors declare the following financial interests/personal relationships which may be considered as potential competing interests: Given his role as Associate Editor, Hao Lu had no involvement in the peer review of this article and had no access to information regarding its peer review. Full responsibility for the editorial process for this article was delegated to another journal editor.
