# Supplementary material for: ELMERF: A deep-learning-assisted hydroponic RGB phenotyping framework for rice seedling salt-stress evaluation and genetic mapping
Source: Plant Phenomics. 2026 Jul 3;8(3):100258. doi: 10.1016/j.plaphe.2026.100258 (PMC13453560; doi:10.1016/j.plaphe.2026.100258)
Supplement: Supplementary file 1 [file mmc1.docx]

**Supplementary Figures**

**Fig. S1.** Relative light intensity at different positions on the cultivation rack (% of maximum light intensity).

**Fig. S2.** Correlation analysis of seedling death rate in different replications under salt stress. Each point represents a single rice genotype, and SYR is expressed as a percentage. *** Significance levels: P < 0.0001.

**Fig. S3.** Correlation analysis of phenotypic trait measurements obtained from RPS and Photoshop. (a) Yellow shoot tissue area. (b) Green shoot tissue area. (c) Root area. Each point represents one sample. The red line and shaded area indicate the fitted regression line and 95% confidence interval, respectively. All area values are expressed in cm².

**Fig. S4.** Representative seedling images. The figure shows 10 images of seedling phenotypes with different salt damage gradients and the corresponding seedling death rate (SDR) and shoot yellowing rate (SYR) values.

**Fig. S5.** Correlation analysis of salt tolerance based on different phenotypic indicators. (a) Correlation between shoot yellowing rate and seedling death rate under salt stress. (b) Correlation between standard evaluation score and seedling death rate under salt stress. *** Significance levels: P < 0.0001.

**Fig. S6.** Consistency analysis between the ratio-based phenotypic traits extracted by ELMERF and the RicePhenoSeg (RPS) ground truth. (a) Correlation scatter plot for yellow shoot tissue ratio. (b) Correlation scatter plot for shoot tissue pixel ratio. (c) Bland–Altman plot for yellow shoot tissue ratio. (d) Bland–Altman plot for shoot tissue pixel ratio.

**Fig. S7.** Quantile-quantile (Q–Q) plots for the GWAS results. Panels (a-c) correspond to the GWAS results for standard evaluation score (SES), seedling death rate (SDR), and shoot yellowing rate (SYR) shown in Fig. 7a-c, respectively. The expected and observed -log₁₀(*p*) values are shown on the x- and y-axes, respectively. The red diagonal line indicates the expected distribution of *p*-values under the null hypothesis.

**Fig. S8.** Representative challenging regions in the RSSD-A. (a–b) Two representative rice seedling images from the RSSD-A. Red boxes indicate local regions with high segmentation difficulty. (1–8) Enlarged views of the corresponding boxed regions.

**Fig. S9.** Representative samples showing variation in foreground class proportions among different images.
